# Metal–Organic Framework‐Based Stimuli‐Responsive Systems for Drug Delivery

**DOI:** 10.1002/advs.201801526

**Published:** 2018-11-20

**Authors:** Wen Cai, Junqing Wang, Chengchao Chu, Wei Chen, Chunsheng Wu, Gang Liu

**Affiliations:** ^1^ Institute of Medical Engineering Department of Biophysics School of Basic Medical Sciences Xi'an Jiaotong University Health Science Center Xi'an Shaanxi 710061 China; ^2^ State Key Laboratory of Molecular Vaccinology and Molecular Diagnostics & Center for Molecular Imaging and Translational Medicine School of Public Health Xiamen University Xiamen 361102 China

**Keywords:** cancer therapy, drug delivery, metal–organic frameworks (MOFs), nanoparticles, stimuli responsive

## Abstract

With the rapid development of nanotechnology, stimuli‐responsive nanomaterials have provided an alternative for designing controllable drug delivery systems due to their spatiotemporally controllable properties. As a new type of porous material, metal–organic frameworks (MOFs) have been widely used in biomedical applications, especially drug delivery systems, owing to their tunable pore size, high surface area and pore volume, and easy surface modification. Here, recent progress in MOF‐based stimuli‐responsive systems is presented, including pH‐, magnetic‐, ion‐, temperature‐, pressure‐, light‐, humidity‐, redox‐, and multiple stimuli‐responsive systems for the delivery of anticancer drugs. The remaining challenges and suggestions for future directions for the rational design of MOF‐based nanomedicines are also discussed.

## 1 Introduction

Smart materials have recently attracted much attention in the field of biomedicine.^1^ In particular, the development of nanotechnology has resulted in much progress in stimuli‐responsive drug delivery systems. Triggers from inside (e.g., internal cues in the cancer microenvironment) and outside (external stimuli) the body provide spatially and temporally controllable characteristics to release the drugs encapsulated in these systems.^2^, ^3^, ^4^, ^5^, ^6^

**Figure 1:**
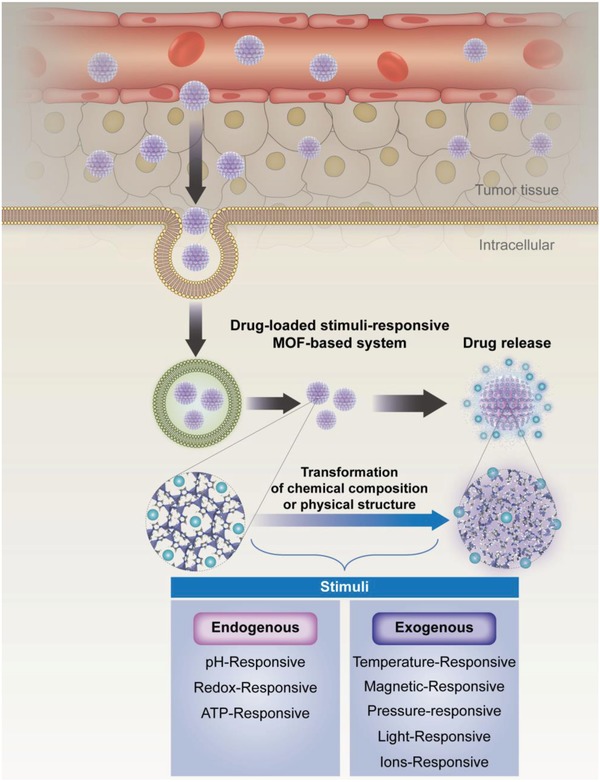
Schematic illustration of metal–organic frameworks (MOFs)‐based stimuli‐responsive system for drug delivery.

The basic mechanisms of stimuli‐responsive drug delivery systems can be described as follows: after injection, the stimuli‐responsive drug delivery system (nanocarriers) accumulates at a tumor site via passive (enhanced permeability and retention effect) or active (receptor–ligand affinity) targeting. When activated by specific triggers, the chemical composition or physical structure of these systems undergoes a given transformation, which induces drug release at the intended site in a controllable fashion (**Figure** **1**). These triggers are generally categorized as intrinsic or external. Intrinsic stimuli are localized within the tumor microenvironment. Compared with healthy tissues, tumor microenvironments have special characteristics such as lower pH and higher temperature that can be employed for developing internal (e.g., pH, redox, ATP) stimuli‐responsive drug delivery systems. Differing from the intrinsic systems utilizing the characteristics of the tumor microenvironment, external (e.g., magnetic field, temperature, ions, pressure, light) stimuli‐responsive systems visualize the nanoparticles (NPs) accumulated at the tumor site and then at the desired time, activate the nanocarriers via triggers from outside the body. External stimuli‐responsive systems provide better spatiotemporal drug release and have demonstrated greater potential for clinical application.

Metal–organic frameworks (MOFs), a new class of porous materials, have been widely investigated for drug delivery in recent years,^7^, ^8^, ^9^, ^10^ due to their high drug‐loading capacity,^10^ easy functionalization,^11^ good biodegradability, and good biocompatibility.^12^, ^13^, ^14^ Among the different stimuli‐responsive drug delivery systems, MOF‐based systems have attracted great interest for achieving a controllable drug release (Figure 1).^14^, ^15^, ^16^, ^17^, ^18^ In this review, we will introduce a variety of MOF‐based stimuli‐responsive systems, including those responding to a single stimulus and multiple stimuli, which provide for the controllable delivery of their loaded drugs upon activation by endogenous (i.e., pH, redox, and ATP) or exogenous (i.e., magnetic field, temperature, ions, pressure, light, and humidity) stimuli.

## 2 Single Stimulus‐Responsive MOFs

### 2.1 pH‐Responsive MOFs

Among the MOF nanocarriers triggered by external stimuli, the pH‐responsive MOFs are the most widely investigated due to the acidic tumor microenvironment and the sensitivity of the coordination bonds in MOFs to external pH.^19^ Many pH‐responsive MOFs have been reported for drug delivery.

#### 2.1.1 Zinc‐Based MOFs

Zeolitic imidazolate framework‐8 (ZIF‐8) is the most commonly used MOF for pH‐responsive drug release; it possesses a sodalite (SOD)‐type structure with large pores (11.6 Å), and due to its porosity and acid sensitivity, shows improved drug loading capacity and pH‐responsive drug release. The polyacrylic acid@ZIF‐8 (PAA@ZIF‐8) nanocarrier was synthesized via ion exchange between Zn^2+^ ions and polyacrylic acid sodium salt and a subsequent reaction with 2‐methylimidazole in a methanol solution.^20^ The PAA@ZIF‐8 nanocarrier has a high doxorubicin (DOX; an anticancer drug) loading capacity of 1.9 g g^−1^ NPs due to electrostatic interactions between the negatively charged PAA and positively charged DOX and the coordination interaction in Zn^2+^–DOX. A confocal laser scanning microscopy analysis showed that the DOX‐loaded PAA@ZIF‐8 released the drug faster in a buffer solution at pH 5.5 than at neutral pH 7.4. These pH‐responsive drug nanocarriers were taken up by MCF‐7 cells via endocytosis and exhibited a low toxicity in the live cells, demonstrating their potential for biomedicine applications.

**Figure 2:**
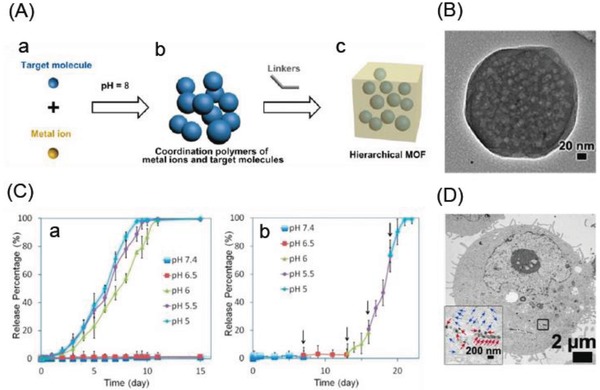
A) The pH‐induced one‐pot synthesis of MOFs with encapsulated target molecules. B) Distribution of mesopores in DOX@ZIF‐8 particles illustrated by TEM image of a DOX@ZIF‐8 single crystal. C) The pH‐responsive release of DOX from DOX@ZIF‐8 particles determined by UV–vis spectrophotometry: a) The typical release system, and b) the stepped release system. D) TEM image of an MDA‐MB‐468 cell (the inset is an enlarged image of the area marked by the square showing individual ZIF‐8 particles (blue arrows) and their aggregates (red arrows)). Reproduced with permission.^23^ Copyright 2016, American Chemical Society.

Tsung and co‐workers^21^ reported on pH‐responsive and fluorescein camptothecin (CPT)‐loaded ZIF‐8 NPs (70 nm). The negatively charged CPT molecules were successfully loaded in the ZIF‐8 NPs via a one‐pot encapsulation route through electrostatic interactions. The pH‐responsive drug release was achieved by the dissociation of the ZIF‐8 framework when in an acidic environment. Cells treated with the CPT‐loaded ZIF‐8 NPs exhibited increased cell death compared with those treated with free CPT. Alsaiari et al.^22^ reported the CC‐ZIFs NPs (ZIF‐8 coloading protein (Cas9) and single guide RNA (sgRNA)) for pH‐responsive drug delivery. The CC‐ZIFs NPs were synthesized by simply mixing Cas9 and sgRNA at 1:1 molar ratio in phosphate buffered saline (PBS) followed by addition of 2‐methylimidazole and zinc nitrate. Cas9 was labeled with Alexa fluor 647 (AF) for pH‐responsive release investigation. The AF‐CC‐ZIFs NPs demonstrated a stable structure under neutral conditions, but started to degrade at acidic pH and completely dissociated in 6 h at pH 5.5. Less than 3% of AF‐Cas9 was released at physiological conditions (pH 7.4), but 60% and 70% were released in 10 min at pH 6 and 5, respectively, attributed to the dissolution of ZIF‐8 at low pH. These CC‐ZIFs NPs demonstrated enhanced endosomal escape resulting in 37% reduction in gene expression over 4 d. Similarly, Zheng et al.^23^ described a one‐pot route for the preparation of ZIFs (ZIF‐8 and ZIF‐67) to encapsulate high loading amounts (14–20 wt%) of organic molecules (DOX and dyes, including rhodamine B, methyl orange, and methylene blue; **Figure** **2**A). Transmission electron microscopy (TEM) demonstrated that the well‐defined molecule‐filled mesopores were homogeneously distributed within the ZIF‐8 crystals (Figure 2B). The DOX‐loaded ZIF‐8 crystals were an efficient nanoplatform for pH‐responsive drug release, retaining the drug under physiological conditions (PBS, pH 7.4), but releasing it in a controlled manner (over 7−9 d) when at low pH (5.0−6.5), which causes ZIF‐8 decomposition (Figure 2C). TEM imaging confirmed the intracellular uptake of ZIF‐8 nanocrystals into MDA‐MB‐468 cancer cells (Figure 2D). Furthermore, the efficacy of the DOX‐loaded ZIF‐8 crystals on breast cancer cell lines was shown to be higher than that of free DOX.

Zou et al.^24^ synthesized ZIF‐8‐coated hollow mesoporous silica NPs (HMSNs; HMSN@ZIF‐8) to produce a pH‐responsive drug delivery system. ZIF‐8 was used both as a self‐sacrificial template to synthesize the HMSNs and as a mesopore blocker grafted onto the HMSNs. The amount of DOX loaded into the HMSNs was as high as 503.4 mmol g^−1^, much more than can be loaded in MCM‐41 mesoporous silica particles. Drug release from the DOX‐loaded HMSN@ZIF‐8 NPs was examined in PBS at pH values of 3.0, 4.0, 5.0, 6.0, and 7.4. At neutral pH (7.4), less than 5% of the DOX was released over a long period, demonstrating the good storage and sealing effects of the ZIF‐8 coating. However, in the acidic environment, a burst release was observed for 60 h. The amount of DOX released (17.1%, 46.6%, 71.4%, and 85%) increased as pH was reduced (6.0, 5.0, 4.0, and 3.0, respectively). These results indicate that HMSN@ZIF‐8 can be an efficient pH‐responsive drug delivery system.

Tian et al.^25^ constructed fluorescein‐loaded graphene oxide‐coated ZIF‐8 nanocrystals (fluorescein‐ZIF‐8/GO) with a fluorescein loading capacity of 1.0 wt%. The fluorescein‐ZIF‐8/GO nanocrystals demonstrated pH‐controlled release behavior. After 10 h, only 6% of the encapsulated fluorescein was released in solutions at pH 7.4, but the rate of release increased dramatically when immersed in an acidic environment due to the dissociation of the ZIF‐8 framework. After 10 h, approximately 80% and 98% of the loaded fluorescein was released in pH 6.0 and 4.5 solutions, respectively. Furthermore, the fluorescein‐loaded ZIF‐8/GO nanocrystals demonstrated excellent photothermal effects under near infrared (NIR) irradiation (808 nm), killing breast cancer 4T1 cells with a high efficacy.

Tang et al.^26^ prepared a thin film coating on ZIF‐8‐NP templates composed of Fe^3+^‐catechol complexes derived from the coordination between Fe^3+^ ions and dopamine‐modified alginate. The pH‐responsive nanocapsule was produced by removing the ZIF‐8 template. DOX was first immobilized in ZIF‐8 via a one‐step coprecipitation during ZIF‐8 synthesis and then loaded in the nanocapsule after the ZIF‐8 was removed. Due to ZIF‐8's large surface area and high porosity and the complexation of Zn^2+^–DOX, the ZIF‐8 DOX‐loading capacity was about 51.3 mg g^−1^. The DOX‐loaded nanocapsules showed an obvious pH‐responsive release under physiological conditions; over 37 h, less than 23% of the DOX was slowly released at pH 7.4, but there was a notable increase at pH 5.0, with 71% released. This investigation provided a new route for preparing pH‐responsive nanocapsules for controlled drug delivery.

By integrating the advantages of NaYF_4_:Yb^3+^, Er^3+^ NPs (UCNPs), a multifunctional upconversion nano‐MOF (NMOF)‐based drug carrier (UCNP@ZIF‐8/FA) was recently constructed with UCNPs, and ZIF‐8 modified with folic acid (FA) as a targeting ligand to provide for simultaneous targeted cellular imaging and pH‐responsive drug release.^27^ The amount of the anticancer drug 5‐fluorouracil (5‐FU) that could be loaded into this upconverted NMOF was 685 mg g^−1^. In vitro 5‐FU release was tested in a pseudo‐physiological pH media (PBS at pH 7.4) and lysosomal pH media (pH 5.5) at 37 °C. After 12 and 24 h at pH 7.4, the loaded UCNP@ZIF‐8/FA carrier had released 35% and 41.5% of the 5‐FU, respectively, whereas 71% and 82% of the 5‐FU was released at pH 5.5 due to the ZIF‐8 shell decomposition in the acidic environment. As a result of folate receptor‐mediated endocytosis, these 5‐FU‐loaded UCNP@ZIF‐8/FA nanocarriers demonstrated great cytotoxicity on HeLa cells.

He et al.^28^ described the construction of multifunctional Fe_3_O_4_@Carbon@ZIF‐8 (FCZ) hybrid NPs for drug delivery. These hybrid NPs not only showed an improved DOX loading capacity of 0.73 g g^−1^ NPs (due both to the encapsulation of ZIF‐8 and the hydrogen bonding between the FCZ NPs and DOX), but also exhibited an excellent pH‐responsive release of the DOX in vitro. The amount of DOX released was considerably larger at an acidic pH (5.5) than at a physiological pH (7.4), with a faster and sustained release of 94.7% at pH 5.5, but only 37.7% under the neutral conditions. In addition, the carbon dots embedded in the porous carbon shell and Fe_3_O_4_ nanocrystals could simultaneously serve as intracellular fluorescence imaging and T_2_‐weighted magnetic resonance imaging (MRI) contrast agents, respectively.

Other zinc‐based pH‐responsive MOFs for drug release have also been reported. Chowdhuri et al.^29^ presented a carboxymethyl chitosan‐modified magnetic NMOF (IRMOF‐3) composed of Zn^2+^ ions and 2‐amino terephthalic acid with a target molecule (folic acid) on its surface. The carboxymethyl chitosan increased the drug loading efficiency and improved the performance of the pH‐responsive drug release. DOX was incorporated into this magnetic NMOF with a loading capacity of 1.63 g g^−1^. DOX release was investigated in vitro in a pseudo‐physiological pH aqueous media (PBS at pH 7.4) and in an intercellular cancer cell environment (pH 5.5) at 37 °C. After 24 h at pH 7.4, ≈26.72% of the DOX was released, whereas at pH 5.5, 55.1% was released. Carbon dots were also encapsulated into this magnetic NMOF for optical imaging. Furthermore, compared with L929 normal cells, this folate‐targeted magnetic NMOF produced more specific cellular internalization in folate‐overexpressing HeLa cancer cells.

A series of new zinc compound coordination polymers with the drug ibuprofen (IBU) incorporated as a ligand have also been reported.^30^ These coordination polymers with MOF‐like structure have a high IBU loading capacity (61 wt%) and demonstrate low cytotoxicity, high stability, high biocompatibility, and good biodegradability. These coordination polymers also showed pH‐controlled IBU release via an ion exchange mechanism.

#### 2.1.2 Iron‐Based MOFs

An iron‐based MOF composed of Fe^2+^ ions and 1,1′‐(1,4‐butanediyl)bis(imidazole) (bbi) was made for encapsulating DOX in situ by simply introducing it into the bbi ligand solution.^31^ The sensitivity of the MOF coordination bonds to external pH allowed this DOX‐encapsulating MOF to release its load in acidic environments. Due to rapid decomposition at low pH, the surface of the MOF was coated with a layer of silica to control the DOX release. Furthermore, FA was conjugated to its surface to allow the nanocomposite to be selectively taken up by cancer cells. This FA‐conjugated and pH‐responsive drug carrier has great potential for applications in drug delivery and cancer therapy. Kim et al.^32^ reported the mussel adhesive proteins (MAPs)‐based polymeric NPs (80–130 nm) containing Fe(III)‐3,4‐dihydroxyphenylalanine (DOPA) complex with MOF‐like structure for the pH‐responsive drug (DOX) release. These MAP‐based Fe(III)‐DOPA NPs demonstrated significant increase of DOX release in a buffer at pH 6 in comparison with other buffers at higher pH values, attributed to the pH‐responsive coordination bonds between Fe(III) and DOPA. These NPs showed effective cytotoxicity on cancer cells through efficient cellular uptake and cytosolic release, and could be further utilized for drug/gene delivery.

Wang et al.^33^ constructed the novel Fe_3_O_4_@C@MIL‐100(Fe) nanocomposite for the synchronous delivery of hydrophobic dihydroartemisinin (DHA) and Fe(III) for cancer therapy. The hydrophobic–hydrophobic interaction between DHA and the hydrophobic benzene rings of MIL‐100(Fe) resulted in a DHA loading capacity as high as 804.9 mg g^−1^. Upon reaching the tumor sites, this delivery system demonstrated a pH‐responsive biodegradation (MIL‐100(Fe) dissociates in acidic solutions) with the synchronous release of DHA and Fe(III). The released Fe(III) was further reduced to Fe(II) and interacted with the released DHA to increase its cytotoxicity. These NPs were taken up by a tumor under the guidance of a magnetic field, and the treatment led to a complete cure with no observable side effects. This system may be a potential alternative antitumor modality in the clinical setting.

Wang et al.^34^ also fabricated core–shell PB@MIL‐100(Fe) dual MOFs (d‐MOFs). The inner PB MOF and outer MIL‐100(Fe) MOF allowed these d‐MOFs to function as fluorescence optical imaging and T1–T2 dual‐modal MRI contrast agents. The hydrophobic anticancer drug artemisinin (ART) showed a high loading capacity of 848.4 mg g^−1^ in this d‐MOF. Due to the pH‐responsive degradation of the outer MIL‐100(Fe) MOF in the low pH lysosomes of tumor cells, the ART was released upon tumor cellular endocytosis. A small amount of ART was released at pH 7.4, but the amount increased at pHs 6.2 and 5.0. The inner PB MOFs' strong absorbance in the NIR region also meant it could be used for photothermal therapy. In vivo photothermal and chemotherapy was performed with an effective tumor ablation in an animal tumor model under the guidance of dual‐modal imaging.

Yang et al.^35^ prepared an intelligent magnetic MOF nanocomposite for the pH‐responsive release of glycoproteins. This MOF nanocomposite was synthesized by modifying Fe_3_O_4_ NPs with a layer of polyvinyl pyrrolidone (PVP) and polyetherimide (PEI) to obtain Fe_3_O_4_@PVP‐PEI nanospheres. Fe^3+^ ions and the organic ligand 1,4‐phenylenebisboronic acid (PBA) were then added to produce the MOF nanocomposite Fe_3_O_4_@PVP‐PEI@MOF‐PBA. The PBA also served as a functional molecule in the MOF–PBA shell. Due to this unique shell, the nanocomposite selectively and pH‐responsively captured and released glycoproteins under moderate pH conditions, capturing at pH 7 and releasing at pH 9. The electron density on the boron atom in the PBA was influenced by the coordinated electron loss from the iron ions, and thus changed PBA's p*K*a. This nanocomposite exhibits great potential for glycoprotein separation.

Zhu et al.^36^ reported that DOX was successfully loaded into the MIL‐100(Fe) shell of the core–shell structure polypyrrole(PPy)@MIL‐100(Fe) with a 12.8% loading ratio. This was possible due to the high porosity of the MIL‐100(Fe) shell, electrostatic interaction between DOX and the carboxylic acid groups in the MIL‐100(Fe) shell, and the coordination bonding of Fe–DOX. The PPy@MIL‐100(Fe) composite demonstrated a pH‐responsive drug release, which was studied in PBS buffers with different pH values (7.4 and 5.0) at 37 °C. Around 42.7% of the DOX was released after 24 h in the neutral PBS (pH 7.4), whereas the gradual degradation of the MIL‐100(Fe) framework under acidic conditions allowed 82.7% to be released at pH 5.0. Furthermore, the PPy core could be used as an organic photothermal therapy agent to improve therapy efficacy. The Fe_3_O_4_‐NH_2_@MIL‐101(Fe)‐NH_2_ magnetic core–shell NPs with sizes ranging from 140 to 330 nm were prepared by microwave irradiation using Fe^3+^ and 2‐amino‐1,4‐benzenedicarboxylate as the metal ions and ligands, respectively.^37^ These magnetic core–shell MOFs had an excellent magnetic response and a high DOX loading capacity of 36.02%. The release of DOX from the loaded magnetic MOF composite could be adjusted by changing the pH. Only a slow and partial release occurred in simulated body fluid at pH 7.4, but because MIL‐101(Fe)‐NH_2_ decomposes under acidic conditions, 53.9% of the DOX was released within 8 h in a simulated tumor cell microenvironment (pH 6.5).

#### 2.1.3 Zirconium‐Based MOFs

A pH‐responsive zirconium‐based MOF (UiO‐66) was applied to the delivery of alendronate (AL).^38^ A high AL loading capacity of 1.06 g g^−1^ was achieved due to the inherent drug anchorages of the Zr–O clusters. The release of AL from loaded UiO‐66 was tested in PBS buffer at 37 °C. Over 60 h, approximately 59% and 42.7% of the DOX was released at pHs 5.5 and 7.4, respectively. This was possible because AL is protonated in acidic environments, which weakens the interaction between AL and the Zr–O clusters in UiO‐66. However, because UiO‐66 has a lower degradation rate under acidic conditions than under neutral and basic conditions,^39^ after 108 h, the amount of AL released at pH 7.4 was more than that released at pH 5.5. The AL‐loaded UiO‐66 inhibited the growth of cancer cells more efficiently than free AL, indicating it is a potential drug carrier for therapeutic applications.

Recently, a cationic NMOF (ZJU‐101) built from zirconium and 2,2′‐bipyridine‐5,5′‐dicarboxylate was reported by the Qian and co‐workers for the delivery of the anionic drug diclofenac sodium (DS).^40^ This positively charged NMOF demonstrated a high DS loading capacity of 0.546 g g^−1^ and a pH‐sensitive drug release. The release of DS was more rapid in PBS at pH 5.4 (inflamed tissues) than at pH 7.4 (normal tissues), because ion exchange between the drug and anions occurs more frequently in acidic environments, which discharges the coulombic interaction between the positively charged ZJU‐101 and negatively charged drug. This pH‐responsive DS‐loaded ZJU‐101 NMOF is a promising candidate for anti‐inflammatory drug delivery.

Qian and co‐workers also experimented with a low cytotoxic porphyrin‐based MOF (PCN‐221) for use as an oral drug carrier that was synthesized using ZrCl_4_ and 5,10,15,20‐tetrakis(4‐carboxyphenyl)porphyrin.^41^ A high methotrexate (MTX)‐loading capacity of 0.40 g g^−1^ was achieved by immersing the PCN‐221 in a solution with the anticancer drug. This MTX‐loaded PCN‐221 demonstrated a controlled pH‐sensitive release of the MTX without a burst effect under physiological conditions. The MTX‐release experiments were performed in PBS buffer solutions at pHs 7.4 (close to the intestinal pH) and 2.0 (≈pH of the stomach) at 37 °C. At pH 2.0, only ≈40% of the loaded MTX was released after 72 h, but 100% was released at pH 7.4 in the same time frame. This pH‐sensitive PCN‐221 MOF is thus a promising oral delivery carrier.

Lázaro et al.^42^ described 200 nm zirconium terephthalate UiO‐66 NPs that were coated with functionalized modulators, loaded with the drug calcein, and surface modified with polyethylene glycol (PEG) chains. At pH 7.4, the PEG chains enhanced the stability of the MOF toward phosphates and overcame the burst release by blocking interaction with the NP exteriors, but at pH 5.5, a stimuli‐responsive drug release occurred.

#### 2.1.4 Gadolinium‐Based MOF

Mechanical grinding was used to transform the gadolinium(III)‐based MOF Gd‐pDBI [pDBI, 1,4‐bis(5‐carboxy‐1H‐benzimidazole‐2‐yl)benzene] to the nanosized MG‐Gd‐pDBI (≈140 nm), which had excellent water dispersibility.^43^ MG‐Gd‐pDBI showed low blood toxicity and a higher DOX loading capacity (12 wt%) than Gd‐pDBI due to its enhanced surface exposure to DOX. The DOX‐loaded MG‐Gd‐pDBI exhibited a pH‐responsive drug release behavior, with only 22% of the loaded DOX released after 5 d at pH 7.4, while 44% was released at pH 5, demonstrating MG‐Gd‐pDBI's potential as a pH‐responsive drug carrier.

#### 2.1.5 Europium‐Based MOF

Duan et al.^44^ developed an antigen‐loaded MOF for cancer therapy that was self‐assembled by Eu^3+^ ions and guanine monophosphate. The maximal encapsulating efficiency of the antigens into this MOF was 55 wt%, and the antigen‐loaded MOF demonstrated pH‐dependent drug‐release behavior. Over 48 h, the antigens were minimally released at pH 7.4, while 60% of the antigens were released at pH 5.0 due to the MOF's dissociation in the acidic environment.

#### 2.1.6 Hafnium‐Based MOF

Nanoscale coordination polymers (NCPs) with NMOF‐like structures are promising carriers for drug delivery. Liu et al.^45^ reported on pH‐responsive NCPs based on high Z element hafnium (Hf) ions and an acidic‐responsive benzoic‐imine linker. These NCPs were modified with PEG for physiological stability and loaded with the chemotherapeutic drug chloro(triphenylphosphine)gold(I) (TPPGC). The TPPGC‐loaded NCP‐PEG NPs dissociated at the lower pH found in tumors due to the acid‐triggered cleavage of the organic linker, which resulted in the release of the TPPGC. The Hf ions could also act as radiosensitizers by absorbing X‐rays for radiotherapy. After the efficient accumulation of TPPGC‐loaded NCP–PEG NPs in a tumor after intravenous injection, a remarkable synergistic therapeutic efficacy was achieved in chemoradiotherapy. These TPPGC‐loaded NCP–PEG NPs are promising agents for the efficient chemoradiotherapy of cancer.

### 2.2 Magnetically Responsive MOFs

Magnetic‐responsive drug delivery is a unique technique using the influence of an external magnetic field, which cannot only precisely drive the drug‐loaded magnetic NPs to the intended site to improve therapeutic efficacy, but also serve as an external stimulus to trigger a controllable release of the drug.^46^, ^47^ More importantly, the MOF‐based magnetic structure could be utilized as a theranostic system for MRI and imaging‐guided therapy.^48^

#### 2.2.1 MOF‐Based Magnetic Core–Shell Structures

MOF‐based nanocarriers for magnetic‐responsive drug delivery are often core–shell structures. In these nanocarriers, magnetic NPs are commonly utilized as a magnetic core with the MOF forming the shell. For instance, a MOF‐based magnetic nanocomposite Fe_3_O_4_@Cu_3_(BTC)_2_ (BTC, benzene‐1,3,5‐tricarboxylate) was constructed by the incorporation of Fe_3_O_4_ nanorods in Cu_3_(BTC)_2_ nanocrystals.^49^ These magnetic nanocomposites exhibited the desirable magnetic performance and high porosity indicative of a promising targeted drug delivery system. The anticancer drug nimesulide (NIM) was encapsulated into this magnetic nanocomposite with a 0.2 g g^−1^ loading capacity by immersing Fe_3_O_4_@Cu_3_(BTC)_2_ in a NIM trichloromethane solution. The loaded NIM was completely released after 11 d in physiological saline at 37 °C.

Guan and co‐workers^50^ reported on the synthesis of γ‐Fe_2_O_3_@MIL‐53(Al) by integrating superparamagnetic γ‐Fe_2_O_3_ NPs into the MOF MIL‐53(Al) via a one‐step in situ pyrolysis route. This magnetic nanocomposite demonstrated a controlled drug release in physiological saline at 37 °C, taking 7 d to completely release the loaded IBU. Furthermore, the superparamagnetic property of γ‐Fe_2_O_3_ endowed this nanocomposite with excellent magnetic performance for targeted drug delivery and separation. Similarly, Wang et al.^33^ described Fe_3_O_4_@C@MIL‐100(Fe) NPs, which showed an increased intracellular accumulation of the drug DHA in tumors under the guidance of an external magnetic field, enabled by the presence of the Fe_3_O_4_.

#### 2.2.2 Magnetic Fe‐MOFs

Sethi et al.^51^ described an iron carboxylate NMOF (F‐NMOF) with high saturation magnetization for magnetically aided drug delivery. The DOX‐loaded F‐NMOF exhibited sustained DOX release in PBS, releasing 88% in 15 d. The cell viability assay demonstrated that the drug‐induced toxicity of the DOX‐loaded F‐NMOF could be further enhanced by use of an external magnetic force, while only negligible magnetic targeting could be achieved with free DOX.

**Figure 3:**
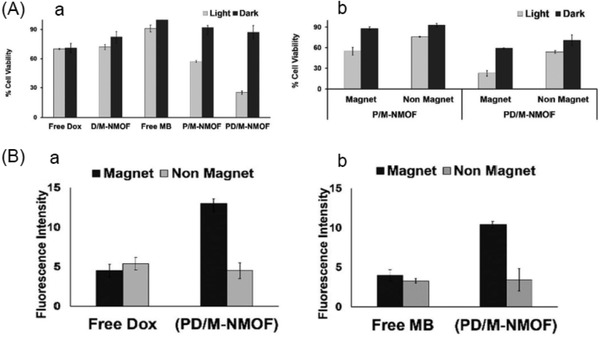
A) Cell (human pancreatic carcinoma cell, Panc‐1) viability of free drugs and loaded drugs, under light irradiation, followed by incubation. B) Enhanced cell (Panc‐1) cytotoxicity of PD/M‐NMOF compared with that of P/M‐NMOF with magnetically aided delivery, followed by light irradiation. The D/M‐NMOF, P/M‐NMOF, and PD/M‐NMOF represented the DOX‐only loaded M‐NMOFs, MB‐only loaded M‐NMOFs, and the MB and DOX co‐loaded M‐NMOFs, respectively. Reproduced with permission.^52^ Copyright 2017, Royal Society of Chemistry.

Similarly, Sharma et al.^52^ described magnetic NMOFs (M‐NMOFs) using iron carboxylate for the magnetically aided codelivery of DOX and the photosensitizer methylene blue (MB; a photodynamic therapy agent) to cancer cells. The drug‐loaded M‐NMOFs exhibited a sustained release of both DOX and MB. In a human pancreatic carcinoma cell (Panc‐1) viability assay, the cytotoxicity of the coloaded M‐NMOFs was much higher than that of the free drugs or with monoloading under light irradiation (**Figure** **3**A). The superparamagnetic behavior of the M‐NMOF allowed the cell cytotoxicity effect of the MB and DOX‐loaded M‐NMOFs to be further enhanced with an external magnetic force (Figure 3B). This integration of magnetic‐field guidance and light‐triggered therapy would improve the efficacy of cancer therapy.

### 2.3 Ion‐Responsive MOFs

Ion‐responsive MOFs have captured much research interest because they offer a new route for drug delivery. In the ion‐responsive MOF‐based drug delivery system, the diffusion and release of drugs is controlled by the strong electrostatic interactions between the ionic drugs and MOFs.

#### 2.3.1 Zn‐MOF

**Figure 4:**
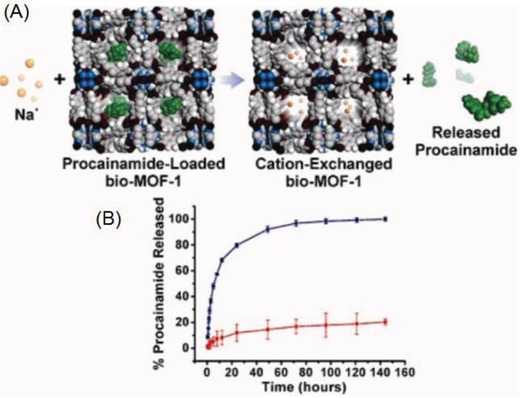
A) Scheme depicting cation‐triggered procainamide release from bio‐MOF‐1. B) Procainamide release profiles from bio‐MOF‐1 (blue, PBS buffer; red, deionized nanopure water). Reproduced with permission.^53^ Copyright 2009, American Chemical Society.

A zinc‐based anionic MOF (bioMOF‐1) was synthesized using biomolecular building blocks by adding biphenyldicarboxylic acid into a mixture of adenine and zinc acetate dihydrate.^53^ The resulting anionic bioMOF‐1 was employed to store procainamide HCl (a cationic anti‐arrhythmia drug), with the electrostatic interaction between the anions and cations producing a loading capacity of 0.22 g g^−1^. The release behavior was investigated by placing procainamide HCl‐loaded bio‐MOF‐1 in PBS buffer (pH 7.4) or deionized nanopure water (control), which showed that the ionic interactions between bio‐MOF‐1 and procainamide allowed drug release to be stimulated by the buffer cations (**Figure** **4**A,B).

#### 2.3.2 Fe‐MOF

Hu et al.^54^ described the positively charged drug carrier MOF‐74‐Fe(III), which was produced via the oxidation of neutral MOF‐74‐Fe(II). An MTT (3‐[4,5‐dimethylthiazol‐2‐yl]‐2,5 diphenyl tetrazolium bromide) assay demonstrated that this cationic MOF had very low cytotoxicity on PC12 cells. IBU anions were loaded into the cationic MOF with an efficient encapsulating capacity (≈15.9 wt%) via ion exchange and salt penetration procedures. Two mechanisms with different drug release rates were involved in the release process. The released at first was mainly sodium IBU and other coordinated free anions with the rate of release governed by the rate of diffusion or ion exchange between the drug‐loaded MOF and PBS solution. The mechanism for the following release of the coordinated drug was triggered by phosphate anions (PO4^3−^) due to their preferential coordination with iron cations. These two distinct kinetic processes made administering the drug release more flexible.

#### 2.3.3 Indium‐Based MOF

Du et al.^55^ effectively controlled the capture and release of drugs by utilizing a preferred coordination strategy. The anticancer drug 5‐fluorouracil (5‐FU) was preferentially captured by MOF‐In1, which consists of In^3+^ ions and tris(para‐carboxylphenyl)phosphine oxide. The size‐ and charge‐selective anion exchange resulted in a loading capacity of 34.32 wt%. As competitive binding sites, the Zn^2+^ ions modulated the 5‐FU release rate from the loaded MOF‐In1. To study the release behavior, the 5‐FU‐loaded MOF‐In1 was immersed in PBS with various Zn^2+^ ion concentrations (500 × 10^−9^ to 10 × 10^−3^ m). Approximately 65% of the 5‐FU was released when the Zn^2+^ concentration was 500 × 10^−9^ m, with more 5‐FU released as the Zn^2+^ concentration increased. Furthermore, because the host−guest interactions between 5‐FU and MOF‐In1 are weakened with increasing temperature, external heating also stimulated the release of 5‐FU. As zinc has a strong influence on the central nervous system, this Zn^2+^ ion ‐triggered drug release system shows promise for the treatment of central nervous system diseases.

### 2.4 Temperature‐Responsive MOFs

#### 2.4.1 PNIPAM@Zr‐MOF

For temperature‐responsive drug delivery systems, variation in the surrounding temperature induces changes in the thermoresponsive materials used, thus regulating the drug release. Among the various thermoresponsive materials, poly(*N*‐isopropyl acrylamide), or PNIPAM, is a well‐known thermoresponsive polymer with a low critical solution temperature. It is hydrophilic and easily dissolves in water when the temperature is below its cloud point (*T*_c_; ≈31 °C); otherwise, it will form an aggregate. Thus, PNIPAM can be used as a building block in thermoresponsive drug delivery systems.^56^ For example, Nagata et al.^57^ described a PNIPAM‐modified MOF (UiO‐66‐PNIPAM) nanocarrier that demonstrated a thermally controllable release driven by simply varying the temperature. The drug‐loaded UiO‐66‐PNIPAM exhibited a quick release of the encapsulated drugs (resorufin, caffeine, and procainamide) at a lower temperature (25 °C), but the release was halted at a higher temperature (40 °C) exceeding its *T*_c_ (31 °C), thus demonstrating the temperature responsiveness.

#### 2.4.2 Zr‐MOFs

Teplensky et al.^58^ developed an optimized treatment route for delaying the release of calcein from two Zr‐based MOFs (NU‐901 and NU‐1000). Crystalline NU‐901 and temperature‐treated NU‐901 showed calcein loading capacities of 37 and 38 wt%, respectively, while the loading capacities of the crystalline and temperature‐treated NU‐1000 were 41.6 and 19.7 wt%, respectively. Temperature treating these two MOFs delayed the release of calcein by 2–7 d compared with their crystalline forms, with complete release not observed until 7 weeks. For example, compared with the crystalline form, the temperature‐treated NU‐1000 delayed the release of calcein by up to 7 d. Although NU‐901 exhibited similar release trends, the effect was not as prominent due to its stronger framework integrity.

**Figure 5:**
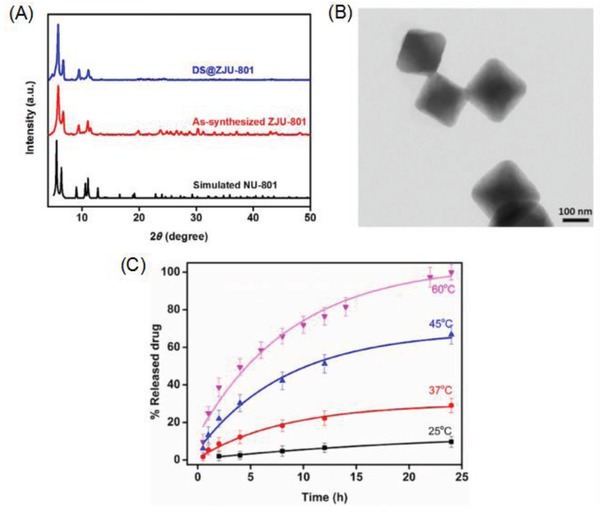
A) PXRD patterns of the synthesized ZJU‐801 and DS@ZJU‐801 along with the simulated NU‐801. B) TEM image of DS‐loaded ZJU‐801. C) DS released from DS‐loaded ZJU‐801 in PBS at 25, 37, 45, and 60 °C. Reproduced with permission.^59^ Copyright 2017, WILEY‐VCH Verlag GmbH & Co. KGaA, Weinheim.

Jiang et al.^59^ designed the Zr‐based MOF ZJU‐801 using (2E,2′E)‐3,3′‐(naphthalene‐1,4‐diyl)diacrylic acid (H_2_NPDA) as an organic linker to produce an exceptional drug delivery system. Due to an appropriate match between the size of DS and the large pore volume of the MOF, it exhibited a high loading capacity of 41.7 wt%. A powder X‐ray diffraction (PXRD) analysis (**Figure** **5**A) shows that ZJU‐801 is isoreticular with NU‐801, which utilize (2E,2′E)‐3,3′‐(1,4‐phenylene)diacrylic acid (H_2_PDA) as organic linker. The size of the DS‐loaded ZJU‐801 NPs was ≈200 nm (Figure 5B). Owing to the bulkier ligand, better stability, and intense π–π interaction between ZJU‐801 and DS, the DS‐loaded ZJU‐801 NPs achieved on‐command heat‐triggered drug release compared with DS‐loaded NU‐801. The release of DS from the loaded ZJU‐801 over 24 h was examined in PBS (pH 7.4) at 25, 37, 45, and 60 °C. The DS‐release rate increased as the temperature increased (Figure 5C), with only negligible releases at 25 and 37 °C, but increased release rates at 45 °C and especially at 60 °C, when the rate was almost 3.4 times faster than that at 37 °C and 10.3 times faster than that at 25 °C. This shows that ZJU‐801 is a promising candidate for temperature‐triggered drug release.

#### 2.4.3 Zn‐MOF

Two porous zinc‐based MOFs (ZJU‐64 and ZJU‐64‐CH_3_) with 1D open channels (1.6 × 1.9 nm) were constructed by Qian and co‐workers from zinc ions, adenine, and carboxylate‐based ligands as a temperature‐responsive drug delivery system.^60^ MTX was successfully loaded into these two MOFs with encapsulating capacities of 13.45 wt% (ZJU‐64) and 10.63 wt% (ZJU‐64‐CH_3_) through a simple impregnation route. The release behavior investigations revealed that it took 72 h at 37 °C for the same amount of MTX to be released from MTX‐loaded ZJU‐64 and ZJU‐64‐CH_3_; however, at 60 °C, it took only 1.5 and 6 h for the MTX to be released from ZJU‐64 and ZJU‐64‐CH_3_, respectively. An MTT assay revealed that these zinc‐based MOFs had low toxicity, and thus potential for biomedical applications. Temperature‐responsive drug delivery by ZJU‐64 and ZJU‐64‐CH_3_ provides a new treatment method to improve therapeutic efficacy by combining drug and thermal therapy.

### 2.5 Pressure‐Responsive MOF

**Figure 6:**
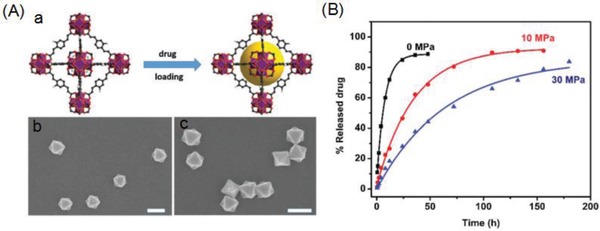
A‐a) Illustration of the topological structure of ZJU‐800 and the process of drug loading and A‐b) SEM image of the as‐synthesized ZJU‐800 and A‐c) SEM image of ZJU‐800 after drug loading. Scale bar = 300 nm. B) DS release from ZJU‐800 with a pressure of 0 MPa (black), 10 MPa (red), and 30 MPa (blue). Reproduced with permission.^61^ Copyright 2016, Royal Society of Chemistry.

Pressure has also been employed for controlling drug release. Qian and co‐workers recently described a zirconium‐based MOF (ZJU‐800) composed of zirconium clusters and (2E,2E′)‐3,3′‐(2‐fluoro‐1,4‐phenylene) diacrylic acid (F‐H_2_PDA) to achieve pressure‐responsive drug release.^61^ The enhanced polarity and extended organic spacer in the MOF allowed DS to be encapsulated with a high loading capacity of 58.80 wt% (**Figure** **6**A). The ZJU‐800 MOF has a homogeneous morphology with an average size of 300 nm both before and after drug loading (Figure 6A), demonstrating its stability. The low cytotoxicity of this MOF was visualized by confocal microscopy and demonstrated with an MTT assay. An adjustable release time of the drug (DS) from 2 to 8 d was achieved by controlling the degree of compaction between the MOF and drug, which applied pressure; the release time was predictably prolonged continuously when the pressure was over 30 MPa (Figure 6B).

### 2.6 Light‐Responsive MOFs

Light‐mediated therapy has been shown to be greatly superior in achieving on‐demand therapeutics and diagnostics in targeted areas both in vitro and in vivo due to its noninvasiveness and spatiotemporal precision by irradiation with a specific wavelength of light.^62^ With the recent developments in nanotechnology, a number of nanomaterials, including NMOFs, have been utilized to construct light‐responsive drug delivery systems.

#### 2.6.1 Zr‐MOFs

**Figure 7:**
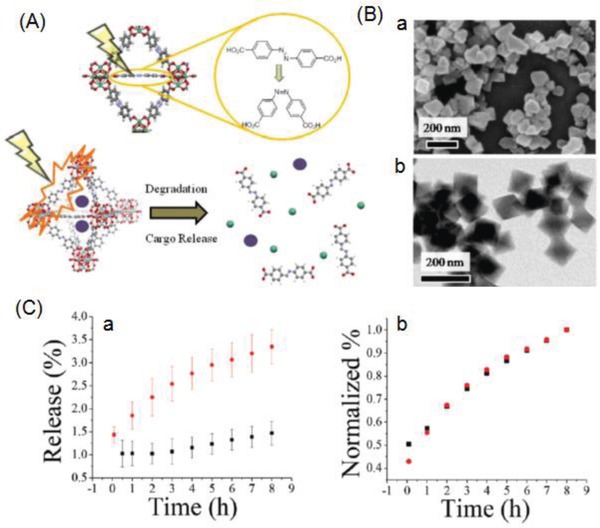
A) Schematic representation of the photodegradation of UiO‐AZB NPs (crystal structure: Zr, green; O, red; C, gray; N, blue; H, white) upon isomerization of the AZB linker from the trans‐form to the cis‐form and subsequent cargo (purple spheres) release. B‐a) SEM and B‐b) TEM image of UiO‐AZB NPs. C‐a) NR release from NR‐loaded UiO‐AZB NPs with no irradiation (black) and with irradiation of 1000W white light (red) and C‐b) normalized degradation of UiO‐AZB NPs (black) and release of NR (red) under irradiation with 1000 W white light. Reproduced with permission.^63^ Copyright 2017, Royal Society of Chemistry.

*Zr‐AZB MOF*: Epley et al.^63^ described the photoinduced degradation of the zirconium‐based MOF UiO‐AZB, employing photoisomerizable 4,4′‐azobenzenedicarboxylate (AZB) as an organic linker, for the drug release. The structure of this NMOF matches that of the UiO‐type framework. UiO‐AZB maintained its structure when the dye molecule Nile red (NR) was loaded, achieving a maximum loading amount of 4.3 wt% (**Figure** **7**A). Scanning electron microscopy (SEM) image (Figure 7B‐a) indicated the average size of UiO‐AZB NP at 107 ± 20 nm, which was additionally confirmed with TEM image (104 ± 12 nm; Figure 7B‐b). The isomerization of the organic struts when irradiated with white light led to the MOF framework degrading and thus releasing the cargo. The release rate of NR from NR‐loaded UiO‐AZB increased ninefold when triggered by irradiation compared with that in the dark (0.36 ± 0.02% h^−1^ vs 0.04 ± 0.01% h^−1^; Figure 7C‐a). This photodegradation‐dominated release mechanism was confirmed by the normalization and overlay of the UiO‐AZB NP degradation and NR release profiles (Figure 7C‐b). This research offers a new route for the development of MOF photocages.

*Zr‐MOF (UiO‐66)*: Nazari et al.^64^ developed a thin layer of MOF (UiO‐66) film on an optical fiber substrate. 5‐FU was encapsulated into the pores of the MOF with a 4 wt% loading capacity by the intramolecular interactions between 5‐FU and UiO‐66, including hydrogen bonding between the oxygen atom of the carbonyl group and the hydroxyl group in the metal‐oxide clusters (O—H···O=C), and the π–π interactions between 5‐FU and the UiO‐66 organic linkers. The delivery of light at an appropriate wavelength through the fiber catheter triggered the 5‐FU to release on demand. At the 1050 nm absorption wavelength, UiO‐66 was activated to overcome the enthalpy of adsorption for 5‐FU and allowed its in situ release, with as much as 110 × 10^−6^ m of 5‐FU delivered within 1 min from one fiber; no 5‐FU was released until the light was delivered. This system provides a new route for local drug administration.

*Zr‐MOF (CORF‐1)*: Diring et al.^65^ constructed a new carbon monoxide (CO)‐releasing framework (CORF‐1) by immobilizing the photoactive manganese carbonyl complex MnBr(bpydc)(CO)_3_ (bpydc = 5,5′‐dicarboxylate‐2,2′‐bipyridine) within a zirconium‐based MOF. The size of the CORF‐1 crystal could be tuned from 260 nm to 1 mm using a coordination modulation method. Efficient and controllable CO‐release from CORF‐1 occurred when it was exposed to low intensity visible light (460 nm), but the release immediately stopped when the light was switched off. In addition, the CO‐releasing properties correlated with the crystal sizes. Moreover, the immobilization of these photoactive crystals in a polymer matrix serving as a cell‐growth substrate allowed for the observation of intracellular uptake of CO upon visible light irradiation to the substrate. This research provides a new opportunity to use MOFs as smart CO‐releasing materials in future therapeutic applications.

#### 2.6.2 PPy@Fe‐MOF

The DOX‐loaded PPy@MIL‐100(Fe) reported by Zhu et al.^36^ also exhibited NIR‐responsive DOX release. Without NIR irradiation, 31.9% (pH 7.4) and 49.1% (pH 5) of the loaded DOX was released after 2 h; however, with NIR irradiation, up to 53.2% (pH 7.4) and 70.4% (pH 5.0) of the DOX was released due to the increased local temperature caused by the photothermal effect of PPy under NIR laser irradiation. The local heating triggered molecular desorption by thermal movement in the crystalline lattice of the carrier as well as by the temperature‐dependent molecular mobility of the drug. This composite shows exceptional promise for synergistic cancer therapy.

#### 2.6.3 Hf‐MOF

The light‐responsive MOFs can be constructed using photosensitizer as the organic links.^66^ For example, the NCPs with MOF‐like structure, which are composed of hafnium ions and bis‐(alkylthio) alkene (BATA; a singlet‐oxygen responsive linker), were utilized as nanocarriers for light‐responsive drug release under red light (660 nm) with a low power density (5 mW cm^−2^).^67^ These NCPs were coloaded with chlorin e6 (Ce6; a photosensitizer) and DOX and then coated with a lipid bilayer that was modified by PEG to obtain NCP–Ce6–DOX–PEG NPs with good colloidal stability. When exposed to light, the singlet oxygen produced from these NPs could be employed not only for photodynamic therapy, but also to induce cleavage of the BATA linker and thus decomposition of the NP structure, thereby releasing the drug. These NCP–Ce6–DOX–PEG NPs produced a chemophotodynamic combination therapy in tumors after their high accumulation, revealed by computed tomography imaging from Hf ions, via intravenous injection. This NCP‐based light‐responsive drug delivery system is a novel theranostic nanoplatform for cancer therapy.

### 2.7 Humidity‐Responsive MOFs

**Figure 8:**
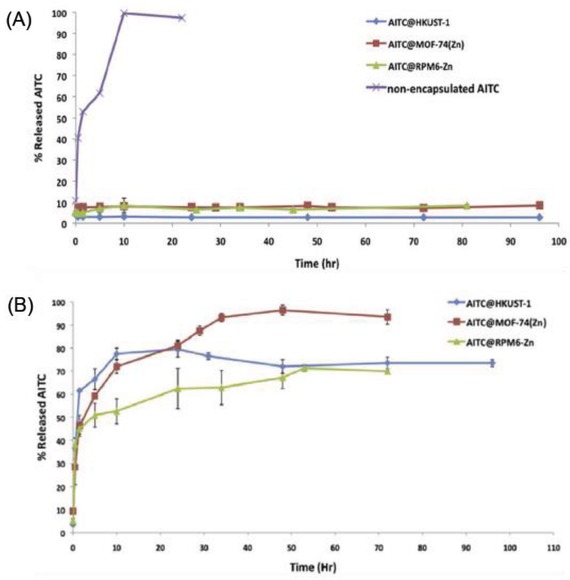
Release profile of AITC molecules from the MOFs (HKUST‐1, MOF‐74(Zn), and RPM6‐Zn) at A) low (30–35%) and B) high (95–100%) relative humidity conditions. Reproduced with permission.^68^ Copyright 2017, Elsevier Ltd.

Humidity‐responsive MOFs present another pathway for drug delivery. Lashkari et al.^68^ described the MOFs HKUST‐1(Cu) (organic linker: 1,3,5‐benzene tricarboxylic acid), MOF‐74(Zn) (organic linker: 2,5‐dihydroxyterephthalate), and RPM6‐Zn (linking ligand: biphenyl‐4,4′‐dicarboxylate; pillar ligand: 4,4′‐azobispyridine) as novel carriers for the high loading (130–400 mg g^−1^ MOF) and controlled release of volatile allyl isothiocyanate (AITC) antimicrobial molecules. The study showed that when the MOFs were exposed to 30–35% relative humidity (RH), only 10% of the loaded AITC was released, with 90% still retained within the pores and channels of the MOFs, while exposing the MOFs to 95% to 100% RH resulted in the release of 70% to 96% of the loaded AITC (**Figure** **8**). This demonstrated that water vapor molecules could trigger the AITC release from these MOFs and that their application for AITC loading and controlled release using high RH as an external stimulus was technically feasible. Since AITC possesses excellent antimicrobial characteristics against a broad spectrum of foodborne pathogens and food spoilage‐inducing microorganisms, especially at low concentrations in the vapor phase, these AITC‐loaded MOFs appear promising for food safety and food industry applications.

### 2.8 Redox‐Responsive MOFs

Redox‐responsive drug delivery is based on the significantly different redox concentrations in tumors and normal tissues. For example, human cancer tissues have significantly higher concentrations of reducing agents such as glutathione (GSH) than normal tissues.^69^, ^70^ The disulfide bond (S—S), a redox‐responsive group, can readily be cleaved in the presence of GSH, making it an attracting receptor site in the construction of redox‐sensitive drug delivery systems.

#### 2.8.1 GSH‐Responsive MOFs

**Figure 9:**
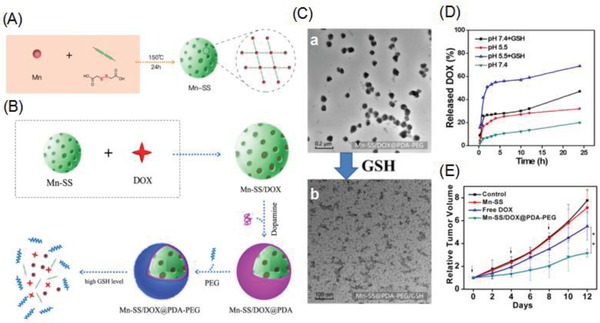
A) Schematic illustration showing the synthesis process of Mn‐SS NPs. B) Scheme demonstrating the preparation of DOX‐loaded NCPs, GSH‐triggered NPs decomposition, and drug release. C) TEM image of a) Mn‐SS/DOX@PDA–PEG NPs and b) Mn‐SS@PDA–PEG 0.25 h after incubation with 10 × 10^−3^ m GSH. D) Cumulative release profiles of DOX from Mn‐SS/DOX@PDA–PEG NPs in PBS with different pH value with or without 10 × 10^−3^ m GSH. E) Tumor growth curves of different groups by various treatments. Reproduced with permission.^71^ Copyright 2017, American Chemical Society.

Liu and co‐workers synthesized GSH‐responsive NCPs composed of manganese ions (Mn^2+^) and dithiodiglycolic acid as the disulfide (SS)‐containing organic linker for drug delivery (**Figure** **9**A).^71^ These Mn–SS NCPs were loaded with DOX via hydrophobic interactions to produce Mn‐SS/DOX NPs, which were then coated with a layer of polydopamine (PDA) and further modified with PEG to yield spherical Mn‐SS/DOX@PDA–PEG NPs (Figure 9B) with a diameter of ≈100 nm (Figure 9C‐a). Owing to disulfide linkage (SS) within dithiodiglycolic acid cleavage in the presence of GSH, the efficient dissociation of these NCPs and thus drug release were achieved. TEM of the Mn–SS/DOX@PDA–PEG NPs before and after being treated with GSH (10 × 10^−3^ m) showed the GSH‐responsive decomposition of the NPs (Figure 9C‐b). The DOX release behaviors from Mn–SS/DOX@PDA–PEG NPs at different pH values with or without GSH were investigated (Figure 9D), which found that the DOX release was remarkably accelerated in the presence of GSH. In addition, the protonation of the amino group on DOX caused the release to become faster at lower pH values. Furthermore, the Mn^2+^ ions in these NCPs provided a strong T1 contrast during MR imaging. These Mn‐SS/DOX@PDA–PEG NPs also exhibited improved in vivo therapeutic efficacy compared to free DOX (Figure 9E). These inherently biodegradable NCPs have the potential for clinical translation.

Similarly, Lei et al.^72^ constructed the novel GSH‐sensitive MOF M‐DTBA (M = Fe, Al, or Zr) using iron (Fe), aluminum (Al), or zirconium (Zr) as metal connecting points and 4,4′‐dithiobisbenzoic acid (4,4′‐DTBA) as the GSH‐sensitive organic bridging ligand. The natural polyphenol anticancer drug curcumin (CCM) was encapsulated into Zr‐DTBA with an 11.8% loading capacity. At a size of 169.4 nm, the CCM‐loaded Zr‐DTBA NPs were suitable for cellular uptake by cancer cells through endocytosis. The disulfide bond in the 4,4′‐DTBA could be cleaved by GSH, leading to the MOF framework's dissociation and thus CCM release. Release from the CCM‐loaded Zr‐DTBA NPs was studied with and without GSH. The release reached 87.4% after 22 h in PBS (pH 7.4) with 10 × 10^−3^ m GSH, while only 50% was released in the same period when GSH was absent. In addition, 85% of the CCM was released in only 5 h at pH 5.5 with 10 × 10^−3^ m GSH, showing the responsiveness of the NPs to the low‐pH tumor microenvironment. These CCM‐loaded Zr‐DTBA NPs demonstrated a much higher antitumor efficacy than free CCM.

#### 2.8.2 Glucose‐Responsive GOx@ZIF‐8

Recently, glucose‐responsive materials have received much attention owing to their ability to competitively combine with glucose, suggesting their great potential in designing drug delivery systems. Duan et al.^73^ developed a novel glucose‐responsive composite based on the MOF ZIF‐8 for self‐regulated insulin release dependent on the glucose concentration. Glucose oxidase (GOx) and insulin were successfully embedded into ZIF‐8 to form the composite insulin‐GOx/ZIF‐8 by mixing aqueous solutions containing Zn^2+^ ions, 2‐methylimidazole, insulin, and GOx. The GOx and insulin loading capacities in this composite were 9.1 wt% and 21.5 wt%, respectively. When a high concentration of glucose is sensed, it enters the pores of the composite and makes contact with the GOx, resulting in glucose oxidation to gluconic acid and H_2_O_2_. The reduced pH in the composite leads to its decomposition and thus insulin release. Investigating insulin release from the ins‐GOx/ZIF‐8 composite at different glucose concentrations found that at 4 mg mL^−1^ glucose, ≈420 µg mL^−1^ of insulin was released in 4 h; however, at either no or 1 mg mL^−1^ glucose, only 84 and 145 µg mL^−1^ were released in 24 h, respectively. These insulin‐GOx/ZIF‐8 composites could be utilized for a glucose‐sensitive and self‐controlling insulin delivery system.

#### 2.8.3 Hydrogen Peroxide (H_2_O_2_)‐Responsive Hf‐MOF

Hydrogen peroxide (H_2_O_2_), the most abundant reactive oxygen species (ROS) molecule produced during ischemia/reperfusion, usually induces inflammation and triggers apoptosis, resulting in oxidative damage to tissues. Thus, targeting H_2_O_2_ as a diagnostic and therapeutic agent holds tremendous potential in cancer therapy. Liu et al. described a H_2_O_2_‐responsive system for drug delivery based on NCPs.^74^ This system was constructed as follows: bovine serum albumin‐stabilized manganese dioxide (MnO_2_) NPs (BM) were coated with NCP shells composed of Hf ions and the cisplatin prodrug c,c,t‐(diamminedichlorodisuccinato)Pt(IV) (DSP); they were then further modified with PEG to form 160‐nm sized BM@NCP(DSP)–PEG NPs. These NPs could be used for radiotherapy with the Hf ions as well as for chemotherapy from the cisplatin released in a reductive environment. The MnO_2_ core in these NPs triggers in situ O_2_ generation from the decomposition of endogenous H_2_O_2_ produced in the cancer cells. The study showed that while adding these NPs to H_2_O_2_ solutions triggered H_2_O_2_ degradation to generate O_2_, without the NPs, the H_2_O_2_ solution was stable, with little spontaneous decomposition. Furthermore, NPs without the MnO_2_ core were also unable to induce O_2_ generation. This generated O_2_ would be helpful for overcoming hypoxia‐associated radio‐resistance. In addition, the MnO_2_ decomposition could lead to enhanced T1 contrast for in vivo tumor MR imaging. This biodegradable theranostic system shows promise as an in vivo combinational chemo‐radiotherapy with great efficacy.

### 2.9 H_2_S‐Responsive MOF

Based on the significantly high content of hydrogen sulfide (H_2_S) in human colon adenocarcinoma cells,^75^ Ma et al.^76^ synthesized a copper‐zinc mixed‐MOF [Cu_2_(ZnTcpp)·H_2_O]*_n_* via a hydrothermal microemulsion reaction between the photosensitive ligand zinc‐metalated 5,10,15,20‐tetrakis(4‐methoxycarbonylphenyl)porphyrin (ZnTcpp) and Cu(NO_3_)_2_·3H_2_O. The Cu^2+^ ions, an important H_2_S‐responding site in H_2_S fluorescence probes, were selected for the metal nodes of this copper‐zinc mixed MOF. The paramagnetic Cu^2+^ ions not only completely quenched the ligand‐based fluorescence, but also significantly minimized the ROS production efficiency of the photosensitive ligand. When H_2_S appeared, the Cu^2+^ ions were removed from the MOF nodes, and thus a luminophor photosensitive ligand was simultaneously obtained. This novel mixed‐metal MOF could be activated by H_2_S in a specific tumor microenvironment for colon adenocarcinoma cancer therapy.

### 2.10 ATP‐Responsive MOF

Adenosine triphosphate (ATP) is a multifunctional nucleotide that provides energy for all biological processes by hydrolyzing phosphoanhydride bonds. Now there is growing evidence indicating that upregulated ATP levels are correlated with many pathological processes including chemoresistance, uncontrolled tumor growth, and synaptic transmission in neurons, making it a significant marker that distinguish cancer cells from normal cells. Inspired by the upregulated ATP levels, researchers have developed many ATP‐responsive drug delivery systems that specifically recognize ATP as well as the competitive binding of ATP aptamers.

Chen et al.^77^ utilized a NMOF comprised of Zr^4+^ ions and amino‐triphenyldicarboxylic acid capped with a complementary nucleic acid (ATP aptamer sequence or the ATP‐AS1411 hybrid aptamer sequence) for drug delivery. In the presence of ATP, these NMOFs were unlocked through the formation of ATP‐aptamer complexes, leading to the loaded drugs being released. MDA‐MB‐231 breast cancer cells treated with the DOX‐loaded ATP aptamer‐gated or ATP AS1411 aptamer‐gated NMOFs for 5 d produced 40% and 55% cell death, respectively, while only 10% of normal MCF‐10A epithelial breast cells died under similar conditions. As ATP is overexpressed in cancer cells and the AS1411 aptamer recognizes the nucleolin receptor sites on their cell membranes, the cytotoxicity was enhanced due to the targeting and effective permeation of the ATP‐responsive NMOFs into the cancer cells.

### 2.11 Competitive Binding Agent‐Responsive MOFs

Supramolecular materials have been extensively studied as their desirable properties such as a special structure, easy functionalization, or desirable host–guest performance.^78^ Modifying nanostructures with these materials offers a new route for potential biomedical applications.^79^ Supramolecular materials such as pillararenes and β‐cyclodextrin (β‐CD) act as gatekeepers and can be utilized to modify MOF surfaces to develop stimuli‐responsive drug delivery systems. When there is a higher binding affinity between a competitive binding agent and a supramolecular material on the surface of a MOF, the release of drugs loaded in the MOF can be triggered by adding the competitive binding agent.

#### 2.11.1 CP5@Zn‐MOF

A monodispersed theranostic nanoplatform (102 nm) with dual stimuli‐responsive (pH and competitive binding agent) drug release capability was constructed based on the zinc‐based MOF UMCM‐1‐NH_2_.^80^ In this work, positively charged Py stalks were attached to UMCM‐1‐NH_2_. Rhodamine 6G (Rh6G) or DOX was then loaded and negatively charged carboxylatopillar[5]arene (CP5), a pillararene‐based supramolecular switch that acts as a gatekeeper, was linked with the Py stalks via host–guest complexation. The addition of a competitive binding agent (methylviologen salts) triggered the drug release because of its higher binding affinity with CP5. In addition, the CP5 tended to be neutral in the acidic tumor environment, which weakened the noncovalent binding interactions between the stalk and CP5, thus releasing the drug. This theranostic nanoplatform exhibits a high drug loading capacity, stimuli‐responsive drug release, low cytotoxicity, good biodegradability, and good biocompatibility, and thus provides a new tool for the controlled release of drugs for cancer treatment.

#### 2.11.2 β‐CD@UiO‐68‐azo

Meng et al.^81^ synthesized an azobenzene‐functionalized Zr‐MOF (UiO‐68‐azo) for loading rhodamine B (RhB) as cargo. The azobenzene units on the surface of the MOF were further bound with β‐CD to construct a stimuli‐responsive mechanized MOF, with the β‐CD ring serving as a gatekeeper to control cargo release. This stimuli‐responsive system demonstrated on‐command cargo release triggered by the addition of competitive agents. A competitive binding agent (e.g., amantadine) with a higher binding affinity to β‐CD triggered the dissociation of the β‐CD rings from the azobenzene stalks and thus released the cargo. In addition, because β‐CD possesses a much higher binding affinity to trans‐azobenzene than to cis‐azobenzene, irradiation by ultraviolet light, which isomerizes azobenzene from trans to cis, also leads to the β‐CD rings dissociating from the azobenzene stalks on the MOF surface and release of the cargo. This MOF‐based system may offer a unique platform for on‐command drug delivery.

### 2.12 Liposome‐Responsive MOFs

Liposomes are known to mimic cell membranes and are considered a true biomimetic system. In the presence of liposomes, drugs loaded in MOFs can be released due to electrostatic interactions between the drugs and liposomes. Adhikari et al.^82^ prepared two DOX‐encapsulated biocompatible MOFs (Fe‐BTC and Zn‐BTC; BTC, 1,3,5‐benzenetricarboxylic acid) via a simple one‐step reaction at room temperature. Compared with conventional routes, this in situ formation of DOX‐loaded MOFs achieved high drug loading capacities (83% and 92% for Zn‐BTC and Fe‐BTC, respectively). In the presence of biocompatible liposomes, the release of DOX absorbed on the surface of the MOFs was triggered by the stronger electrostatic interactions between DOX and the liposomes. In addition, in acidic environments, the carboxylate anions were protonated and the coordination between the carboxylic acid group and metal center broke down, resulting in the release of DOX through the disruption of the MOF. These MOFs provide new pathways for liposome‐responsive drug release.

Similarly, these authors have also described two DOX‐loaded ZIFs (ZIF‐7 and ZIF‐8) for the liposome‐responsive release of DOX.^83^ The loading capacity of DOX into ZIF‐7 and ZIF‐8 was approximately 40% and 52%, respectively, the difference being due to ZIF‐7 having a bigger cavity size than ZIF‐8. This study showed that both ZIFs were excellent for DOX release when in contact with liposomes. In addition, ZIF‐8 also provided for controlled release of DOX as the pH changed from 7.4 to 4.0. However, ZIF‐7 remained intact at the lower pH because it is more stable than ZIF‐8 under acidic conditions. This study suggests the drug release rate could be adjusted by a combination of liposomes and different ZIFs.

## 3 Multiple Stimuli‐Responsive MOFs

Due to the complexity of the environment in the human body, improving the therapeutic efficacy of drug delivery systems usually requires it to be responsive to multiple triggers rather than a single stimulus. Thus, multiple stimuli‐responsive systems based on MOFs have been developed to achieve more precise cancer therapies.

### 3.1 Supramolecular Material‐Based Multiple Stimulus‐Responsive MOFs

#### 3.1.1 Zn^2+^ Ion/Temperature‐Responsive: CP5@Zr‐MOF

Tan et al.^84^ constructed a novel dual stimuli‐responsive system based on a Zr‐MOF (UiO‐66‐NH_2_), which was loaded with 5‐FU and capped with CP5‐based supramolecular switches as gatekeeper to produce CP5@UiO‐66‐NH_2_/5‐FU. In this system, positively charged quaternary ammonium salt (Q) stalks were installed on the surface of UiO‐66‐NH_2_. The drug 5‐FU was then loaded and negatively charged CP5 macrocycles were utilized to encircle the Q stalks via host–guest complexation to form [2]pseudorotaxanes that acted as gates to control the release of 5‐FU. The higher binding affinity between the Zn^2+^ ions and CP5 allows the Zn^2+^ ions (abundant in synaptic vesicles) to stimulate 5‐FU release. External heating was also introduced to regulate the release, because increasing the temperature weakens the noncovalent bonding interactions between the stalks and CP5. This novel theranostic system provides a new avenue for the treatment of central nervous system diseases.

#### 3.1.2 pH/Temperature/Competitive Binding Agent‐Responsive: CP5@Zr‐MOF

Tan et al.^85^ described a nanoplatform based on a Zr‐MOF (UiO‐66‐NH_2_) for multiple stimuli‐responsive drug delivery. In this platform, the Zr‐MOF was modified by positively charged quaternary ammonium salt (A) stalks to form UiO‐66‐NH‐A NPs, which were then capped by encircling the stalks with negatively charged CP5. These NPs were cubic shaped with a size <100 nm. After modification with biocompatible A stalks, the NPs were reduced to 20 nm by sonication, but increased to 40 nm after drug loading and capping. This platform controlled 5‐FU drug release in bone tumor cells with increasing concentration of Ca^2+^, a competitive binding agent. Only 5% of the 5‐FU was released at 1 × 10^−3^ m Ca^2+^. Raising the concentration to 300 × 10^−3^ m resulted in all of the 5‐FU being released within 2 d. Low pH could also release the 5‐FU. It was retained at neutral pH, while approximately 18% and 54% of the 5‐FU was released in 1 h at pHs 5.0 and 4.0, respectively. Last, increasing temperature released the 5‐FU, with no apparent release at 25 or 37 °C, but good release at 60 °C. All of these stimuli (elevated Ca^2+^ concentration, reduced pH, and increased temperature) weakened the supramolecular interactions between the MOF and CP5. This research provides an opportunity to develop platforms for bone regeneration and bone cancer therapy.

#### 3.1.3 pH/GSH‐Responsive: β‐CD‐SS‐BCN@MIL‐101(Fe)

Wang et al.^86^ reported on a MIL‐101(Fe)‐based system for pH‐ and GSH‐sensitive drug release. MIL‐101(Fe) was loaded with DOX and then modified by β‐CD‐SS‐BCN (a β‐CD derivative) and K(ad)RGDS‐PEG1900 (a targeted peptide functionalized polymer). The pH‐responsive benzoic imine bond in K(ad)RGDS‐PEG1900 and the GSH‐responsive disulfide bond after linking β‐CD at the surface of the MOF allowed this system to demonstrate both pH‐ and GSH‐responsive drug release behavior. This system exhibited enhanced cellular uptake in the acidic tumor environment and tumor intracellular GSH‐responsive drug release, effectively inhibiting tumor growth.

### 3.2 Other Multiple Stimuli‐Responsive MOFs

#### 3.2.1 pH/Redox‐Responsive: Fe‐MOF

*pH/GSH‐Responsive: MIL‐100(Fe)*: Tan et al.^87^ reported on MIL‐100(Fe)‐based metal–organic gels (MOGs) for the delivery of DOX, which was loaded into the MOGs with a loading capacity of 0.4 g g^−1^. Because the structural integrity of the MOG was disrupted by acidic or GSH‐containing environments, a significant amount of DOX could be released from the DOX‐loaded MOGs in an acidic pH (5.5) or in the presence of GSH. There was no significant DOX released at pH 7, while the release of DOX was obvious at pH 5.5, with 1 mg mg^−1^ observed. In addition, exposure to GSH produced a significant release of 0.6 mg mg^−1^. In vitro experiments with HeLa cancer cells further demonstrated stimuli‐responsive drug release from the DOX‐loaded MOGs.

*pH/H_2_O_2_‐Responsive: Fe‐EA*: Zhao et al.^88^ synthesized Fe‐EA nanoframework NPs by using the biosafe material ellagic acid (EA), polyvinylpyrrolidone (PVP), and ferric ions as building blocks. The amount of EA in this nano‐framework was determined to be 60 wt% based on the amount of the element Fe measured by inductively coupled plasma‐atomic emission spectrometry. The intracellular degradation of the Fe‐EA NPs in HeLa cells was due to their pH‐ and H_2_O_2_‐responsive properties. These NPs partially degrade in a mildly acidic environment (pH 5.0), providing MR contrast enhancement. In addition, the Fe‐EA NPs acted as a hydrogen peroxide catalyst toward H_2_O_2_. Compared with the control group, the color fading of the Fe‐EA NPs suspended in H_2_O_2_ solution with generated O_2_ bubbles could be observed; this was due to the dissociation of the Fe‐EA frameworks. These biodegradable NPs can provide for effective in vivo imaging‐guided photothermal therapy without apparent toxicity.

#### 3.2.2 pH/Redox‐Responsive: P@ZIF‐8

Zhou et al.^89^ produced a nanocomposite (P@ZIF‐8) for use as a dual stimuli‐responsive drug release system by utilizing a selenium‐containing polymer (P) with redox‐responsive property and the MOF ZIF‐8 with its pH‐sensitive property. In this nanocomposite, the selenium‐containing polymer and ZIF‐8 served as core and shell, respectively. In the presence of a redox agent and pH stimuli, the DOX‐loaded nanocomposite demonstrated dual stimuli‐responsive release capability in vitro. When triggered by GSH (1 × 10^−3^ m), DOX release from the nanocomposite was inhibited at pH 7.4, with only 21.1% released over 24 h due to the blocking effect of the ZIF‐8 shell. However, the release accelerated up to 81.2% at pH 5.0 and 100% at pH 4.2, demonstrating the pH‐responsive property of the ZIF‐8 shell acting as the gatekeeper. A similar release trend was observed when triggered with H_2_O_2_ (1 × 10^−3^ m) or a relatively low concentration of redox agents (0.5 × 10^−3^ m) in solution. Without the redox agents, DOX release was only 5.1% at pH 7.4, 1.6% at pH 5.0, and 14.5% at pH 4.2 after 48 h, indicating the nanocomposite was stable in PBS and that it was the synergistic effect of the pH and redox agents that triggered the DOX release. This nanocomposite is a promising candidate for realizing controlled drug delivery in tumors.

#### 3.2.3 NIR/H_2_O_2_‐Responsive: UCNP/MB@ZIF‐8/Catalase

The core–shell nanocomposite UCNPs/MB@ZIF‐8@catalase (UCNPs, upconversion NPs; MB, methylene blue) was developed for NIR/H_2_O_2_‐responsive photodynamic therapy against hypoxic tumor cells.^90^ The synthesis procedure involved a one‐pot assembly of a UCNPs/MB@ZIF‐8 core–shell structure and the subsequent surface functionalization of the shell with catalase. The loading capacity for MB in this nanocomposite was 1.97 wt%. DPBF (1,3‐diphenylisobenzofuran; absorption peak at 410 nm) was used as a probe to evaluate the extracellular generation of singlet oxygen (^1^O_2_) from this nanocomposite, which occurs because the catalase on the surface catalyzes the breakdown of H_2_O_2_ to generate oxygen. Upon exposure to N_2_ under 980 nm irradiation, only the nanocomposite dispersed in H_2_O_2_‐containnig PBS buffer induced an obvious decrease in the DPBF absorption, demonstrating it could generate ^1^O_2_ in hypoxic cells and improve the efficiency of photodynamic therapy in solid tumors. The high porosity of the ZIF‐8 shell provided an efficient platform for adsorbing the oxygen molecules and promoting ^1^O_2_ generation. The ZIF‐8 shell also prevented the aggregation and leakage of MB molecules and facilitated fluorescence resonance energy transfer from the UCNPs to the MB with a 17.9% efficiency. These results indicate this nanocomposite could be applied for photodynamic therapy as well as bioimaging.

#### 3.2.4 pH/Temperature‐Responsive: Zn‐TBDA

Lin et al.^91^ described the MTX‐loaded MOF Zn‐TBDA (TBDA, 4′‐(1H‐tetrazol‐5‐yl)‐[1,1′‐biphenyl]‐3,5‐dicarboxylic acid), which has 1D channels for pH‐ and temperature‐responsive drug delivery. MTX was loaded into Zn‐TBDA with a 12.59 wt% loading capacity through an in situ encapsulation process. The MTX release rate and amount increased as the pH value was reduced or the temperature increased. Below 37 °C, only 43% of the MTX was released at pH 7.4 over 48 h, but the amount increased to 61% at pH 6.5 due to the influence of the acidic conditions on the MOF structure. In addition, more MTX was released at a faster rate at 42 °C (tumor temperature) than at 37 °C (normal tissue temperature), because the host–guest interactions were weakened by the higher temperature, thus promoting the release of MTX. This means the drug release can be controlled by differences in the internal microenvironments of normal and diseased cells, indicating potential applications for Zn‐TBDA as a smart drug carrier.

#### 3.2.5 pH/K^+^ Ion‐Responsive: Zn‐MOF

Kahn et al.^92^ prepared pH‐ and K^+^ ion‐responsive DNA‐functionalized MOFs consisting of Zn^2+^ ions and two different organic ligands, amino terephthalic acid and 4,4′,4″‐benzene‐1,3,5‐triyl‐tribenzoic acid (BTB), by implementing three different DNA switching motifs, including the i‐motif and triplex DNA nanostructures for the pH‐responsive property as well as a K^+^‐stabilized G‐quadruplex acting as an ion‐gating unit, for the switchable release of the loadings. The dye rhodamine 6G (Rh6G) was loaded into this MOF via π–π and H‐bond interactions between Rh6G and the MOF pores. The stimuli, including pH and K^+^ ions, triggered the controllable release of Rh6G from the MOFs. At pH 7.4, the i‐motif units dissociated, thereby unlocking the MOFs and releasing the loadings, whereas at pH 5.5, the i‐motif structures locked the MOFs. In the presence of K^+^ ions, the switchable ON–OFF cyclic release of Rh6G from the MOFs was triggered by the dissociation of the K^+^‐stabilized G‐quadruplex by means of 18‐crown‐6‐ether and the reassembly of the G‐quadruplex locking units. This stimuli‐responsive DNA/MOF nanocomposite provides a new method for assembling other DNA/MOF nanocomposites that respond to triggers.

#### 3.2.6 ATP/Mg^2+^ Ion‐Responsive: Zr‐MOF

Like the ATP‐responsive NMOF reported in ref., 77 Chen et al.^93^ also reported on a nucleic acid‐functionalized Zr‐MOF for ATP‐ and Mg^2+^ ion‐responsive drug delivery. This Zr‐MOF was loaded with fluorophores or DOX and capped by a metal ion‐dependent DNAzyme as a lock. The presence of Mg^2+^ or Pb^2+^ ions catalyzed the cleavage of the nucleic acid locks, leading to the release of the load. The stimuli‐responsive NMOFs were prepared by conjugating the AS1411 aptamer sequence to the Mg^2+^ ion‐dependent DNAzyme. Unlocking these NMOFs only worked in the presence of ATP and Mg^2+^ ions, which acted as cooperative triggers. When these ATP/Mg^2+^ ion‐responsive NMOFs were loaded with DOX, they demonstrated selective cytotoxicity against MDA‐MB‐231 cancer cells because of the enhanced permeation of the NMOFs into the cells via receptor‐mediated endocytosis (the AS1411 aptamer sequence binds to the nucleolin in cancer cells) and the targeted permeation of the NMOFs into the cells.

## 4 Challenges and Future Prospects

Recently, the use of stimuli‐responsive systems as drug carriers for on‐demand drug release has gained increasing attention around the world, and these systems show great potential for cancer therapy. Stimuli‐responsive NMOFs, a new class of stimuli‐responsive materials, demonstrate great potential in overcoming the limitations and drawbacks of conventional drug delivery systems for controllable spatiotemporal drug release to achieve good therapeutic efficacy (summarized in **Table** **1**).

**Table 1:** Stimuli‐responsive drug delivery systems based on MOFs

Stimuli	Drug delivery systems based on MOFs	Mechanism	Refs.
pH	DOX/PAA@ZIF‐8	pH‐cleavable bonds	20
	CPT/ZIF‐8		21
	DOX/ZIFs		23
	DOX/MSN@ZIF‐8		24
	Fluorescein/ZIF‐8@GO		25
	DOX/Fe^3+^‐catechol		26
	5‐FU/UCNP@ZIF‐8@FA		27
	DOX/Fe_3_O_4_@C@ZIF‐8		28
	DOX/IRMOF‐3@FA		29
	IBU/Zn‐MOF		30
	DOX/Fe‐bbi@Silica@FA		31
	DHA/Fe_3_O_4_@C@MIL‐100(Fe)		33
	ART/PB@MIL‐100(Fe)		34
	Glycoprotein/Fe‐PBA		35
	DOX/PPy@MIL‐100(Fe)		36
	DOX/Fe_3_O_4_@MIL‐100(Fe)		37
	AL/UiO‐66(Zr)		38
	DS/ZJU‐101(Zr)		40
	MTX/PCN‐221(Zr)		41
	Calcein/UiO‐66(Zr)		42
	DOX/Gd‐PDBI		43
	Antigens/Eu‐MOF		44
	TPPGC/Hf‐BI		45
	Rh6G (DOX)/CP5@Zn‐MOF		80
	5‐FU/CP5@Zr‐MOF		85
	DOX/Fe‐BTC(Zn‐BTC)		82
	DOX/ZIFs		83
	DOX/β‐CD@MIL‐100(Fe)		86
	DOX/MIL‐100(Fe)		87
	DOX/P@ZIF‐8		89
	MTX/Zn‐TBDA		91
	Rh6G/DNA@Zn‐MOF		92
Magnetic	NIM/Fe_3_O_4_@Cu_3_(BTC)_2_	Magnetic‐responsive materials	49
	IBU/γ‐Fe_2_O_3_@MIL‐53(Al)		50
	DOX/Fe‐MOF		51
	(MB+DOX)/Fe‐MOF		52
Ions	Procainamide HCl/BioMOF‐1	Electrostatic interactions	53
	IBU anions/MOF‐74‐Fe(III)		54
	5‐FU/MOF‐In1		55
	5‐FU/CP5@Zr‐MOF		84
	Rh6G/DNA@Zn‐MOF		92
	DOX/DNAzyme@Zr‐MOF		93
Temperature	Drugs (resorufin)/PNIPAM@UiO‐66(Zr)	Thermal‐responsive materials	57
	Calcein/Zr‐MOF		58
	DS/ZJU‐801(Zr)		59
	MTX/Zn‐MOF		60
	Rh6G (DOX)/CP5@Zn‐MOF		80
	5‐FU/CP5@Zr‐MOF		84
	MTX/Zn‐TBDA		91
Pressure	DS/Zr‐MOF	Pressure‐responsive materials	61
Light	NR/Zr‐AZB	Photo‐responsive materials	63
	5‐FU/UiO‐66(Zr)		64
	CO/CORF‐1		65
	DOX/PPy@MIL‐100(Fe)		36
	(Ce6+DOX)/Hf‐BATA		67
	RhB/β‐CD@UiO‐68‐AZO		81
	MB/UCNP@ZIF‐8@Catalase		90
Humidity	AITC/Cu‐MOF (Zn‐MOF)	Relative humidity triggers the drug release	68
Redox (GSH, Glucose, H_2_O_2_)	DOX/Mn‐SS	Redox‐responsive dissociation of materials	71
	CCM/Zr‐DTBA		72
	(Ins+GOx)/ZIF‐8		73
	Cisplatin/Hf‐DSP		74
	DOX/β‐CD@MIL‐100(Fe)		86
	DOX/MIL‐100(Fe)		87
H_2_S	[Cu_2_(ZnTcpp)·H_2_O]_n_ (Photosensitive ligand ZnTcpp‐constructed MOF)	H_2_S‐responding site (Cu^2+^ ions)	76
ATP	DOX/Zr‐MOF	Formation of ATP‐aptamer complexes	77
	DOX/DNAzyme@Zr‐MOF		93
Competitive binding agent	Rh6G (DOX)/CP5@Zn‐MOF	Higher binding affinity	80
	5‐FU/CP5@Zr‐MOF		85
	RhB/β‐CD@UiO‐68‐AZO		81
Liposome	DOX/Fe‐BTC (Zn‐BTC)	Electrostatic interactions	82
	DOX/ZIFs		83

Although rapid progress has been made on the study of stimuli‐responsive MOF‐based drug delivery systems, there are still many issues that should be addressed before their clinical application.

(1) Each stimuli‐responsive modality has its own limitations. For example, compared with the external stimuli‐responsive modality, the intrinsic stimuli‐responsive modality (e.g., pH) has low flexibility. However, the external stimuli‐responsive modality also has its drawbacks. For instance, the magnetic‐responsive modality needs a large amount of sample to work and the light‐responsive modality suffers from shallow penetration. Although the multiple stimuli‐responsive modality is developing to overcome these limitations, the research is still in its infancy. More effort should be devoted to the development of MOF‐based multiple stimuli‐responsive drug delivery systems for achieving better therapeutic efficacy.
(2) More studies should be focused on the preparation of MOF‐based stimuli‐responsive drug delivery systems with low‐toxicity and good colloidal stability. The investigations for the synthesis of biocompatible MOF‐based stimuli‐responsive NPs with good stability is still insufficient. To reduce the toxicity of NPs, utilizing “green” synthesis routes and selecting endogenous components as organic linkers to prepare metal biomolecule frameworks (bioMOFs) may be good choices. The colloidal stability of NPs was still a major issue that limited their biomedical applications and this could be improved by surface modification with functional molecules.^94^, ^95^, ^96^
(3) Furthermore, researchers must optimize the performance of MOF‐based stimuli‐responsive drug delivery systems prior to clinical application by conducting systematic in vivo studies on their stability, degradation mechanics, and side effects on normal organs. For example, a lot of researches have reported the in vitro toxicity of MOF NPs, but these in vitro experiments based on established cell lines are hardly reproduced in the in vivo situation; currently, only very few studies on the in vivo toxicity of MOF NPs have been performed.^12^, ^43^ In addition, although there have been emerging reports on MOF NPs for in vivo cancer therapy,^7^ few of the delivery systems were MOFs‐based stimulus‐responsive NPs.^43^, ^44^, ^45^, ^76^, ^86^, ^88^ Most of the studies on MOF‐based stimulus‐responsive NPs for drug delivery were still in the in vitro step, more systematic in vivo investigations should be carried out for preclinical evaluation.

## 5 Conclusions

As a new class of porous materials, MOFs have the advantages of high drug loading capacities, easy functionalization, and good biocompatibility. Thus, a variety of MOF‐based stimuli‐responsive drug delivery systems have been reported and considerable achievements have been made in recent years. However, more work is required to develop multifunctional MOF‐based stimuli‐responsive drug delivery systems. Most importantly, more investigations on their toxicity after long‐term use and in vivo research with these systems should be conducted to determine possible side effects before their clinical translation. In conclusion, the development of MOF‐based stimuli‐responsive drug delivery systems with low toxicity, biodegradability, and high therapeutic efficacy is a promising research direction, but there is still a long way to go before their clinical application.

## Conflict of Interest

The authors declare no conflict of interest.
